# Macrophage Migration Inhibitory Factor Promoter Polymorphisms (−794 CATT_**5–8**_ and −173 G>C): Relationship with mRNA Expression and Soluble MIF Levels in Young Obese Subjects

**DOI:** 10.1155/2015/461208

**Published:** 2015-04-20

**Authors:** Inés Matia-García, Lorenzo Salgado-Goytia, José F. Muñoz-Valle, Samuel García-Arellano, Jorge Hernández-Bello, Aralia B. Salgado-Bernabé, Isela Parra-Rojas

**Affiliations:** ^1^Laboratorio de Investigación en Obesidad y Diabetes, Unidad Académica de Ciencias Químico Biológicas, Universidad Autónoma de Guerrero, 39090 Chilpancingo, GRO, Mexico; ^2^Instituto de Investigación en Ciencias Biomédicas, Centro Universitario de Ciencias de la Salud, Universidad de Guadalajara, 44340 Guadalajara, JAL, Mexico

## Abstract

We analyzed the relationship of −794 CATT_5–8_ and −173 G>C *MIF* polymorphisms with mRNA and soluble MIF in young obese subjects. A total of 250 young subjects, 150 normal-weight and 100 obese subjects, were recruited in the study. Genotyping of −794 CATT_5–8_ and −173 G>C *MIF* polymorphisms was performed by PCR and PCR-RFLP, respectively. MIF mRNA expression was determined by real-time PCR and serum MIF levels were measured using an ELISA kit. For both *MIF* promoter polymorphisms, no significant differences in the genotype and allele frequencies between groups were observed. MIF mRNA expression was slightly higher in obese subjects than in normal-weight subjects (1.38-fold), while soluble MIF levels did not show differences between groups. In addition, we found an increase in MIF mRNA expression in carriers of the 6,6 and C/C genotypes and the 6G haplotype of the −794 CATT_5–8_ and −173 G>C *MIF* polymorphisms, although it was not significant. In conclusion, this study found no relationship between obesity and *MIF* gene promoter polymorphisms with MIF mRNA expression in young obese subjects.

## 1. Introduction

Obesity is a chronic, complex, and multifactorial disease characterized by a state of chronic low-grade systemic inflammation. This chronic inflammation is involved in insulin resistance (IR), which is the underlying condition of type 2 diabetes mellitus (T2DM) and metabolic syndrome [1, 2]. Several studies have shown that obesity is associated with elevated serum levels of a wide range of inflammatory markers including C-reactive protein (CRP), interleukin 6 (IL-6), interleukin 8 (IL-8), and monocyte chemoattractant protein 1 (MCP-1) [3, 4].

Macrophage migration inhibitory factor (MIF) is a protein with a molecular weight of 12.5 kDa [5]; it was one of the first cytokines reported in 1966 and described as a T cell derived cytokine that inhibited the random migration of macrophages* in vitro* and promoted macrophage accumulation during delayed-type hypersensitivity reactions [6, 7]. Since MIF is recognized as a proinflammatory cytokine and obesity is associated with a chronic inflammatory response, MIF may have an impact on the pathophysiology of obesity [5, 8]. MIF is produced by different cells and tissues, including T cells, macrophages, monocytes, pituitary gland, fibroblasts, endothelial cells, and adipocytes [9–11]. In addition, MIF counterregulates the immunosuppressive actions of glucocorticoids and promotes the expression and secretion of proinflammatory mediators such as tumor necrosis factor *α* (TNF*α*), interleukin 1*β* (IL-1*β*), interleukin 2 (IL-2), IL-6, IL-8, and interferon gamma (IFN*γ*) [5, 12, 13].

Previous studies have reported that circulating MIF levels are elevated in rheumatoid arthritis (RA), systemic lupus erythematous (SLE), insulin resistance (IR), and type 2 diabetes mellitus (T2DM). Since these diseases are accompanied by persistent inflammation of varying degrees [14–17], it is important to conduct studies to try to elucidate the role of MIF in disease development.

An increase in soluble MIF levels has also been reported in obese subjects; several epidemiological studies relate circulating MIF levels with increased markers of inflammation and markers of beta-cell dysfunction. Furthermore, it has been observed that physical activity and a dietary-focused weight management program resulted in reduction of MIF levels in obese subjects [18–20].

The* MIF* gene is located on chromosome 22q11.23 and it has been linked with abdominal obesity in Caucasians in a genome-wide linkage scan. This may suggest that this chromosomal region is a susceptibility* locus* for abdominal adiposity in a particular population [21]. Two polymorphisms have been identified in the promoter region relative to the site of transcription with functional importance: (1) the short tandem repeat (STR) −794 CATT_5–8_ MIF (rs5844572), which is a microsatellite repetition of Cytosine-Adenine-Thymine-Thymine (CATT) at position −794 bp, and the repeat length (5 to 8 repetitions) which correlates with increased gene expression and with circulating MIF levels; (2) the single nucleotide polymorphism (SNP) −173 G>C MIF (rs755622) at position −173 of the* MIF* gene with a change from Guanine (G) by Cytosine (C). The −173∗C allele has been associated with mRNA expression and circulating MIF levels [22–24]. In previous reports, both functional MIF polymorphisms have been related with autoimmune/inflammatory pathologies such as RA, SLE, and psoriatic arthritis, as well as obesity and diabetes [15, 22, 25–31].

The aim of this study was to investigate the relationship of −794 CATT_5–8_ and −173 G>C* MIF* polymorphisms with MIF mRNA and soluble MIF expression in young obese subjects.

## 2. Materials and Methods

### 2.1. Subjects

We recruited a total of 250 subjects, 18 to 30 years old, 150 normal-weight subjects and 100 obese subjects from the state of Guerrero, Mexico. Exclusion criteria included acute inflammatory diseases or any medication intake at the time of the investigation. All subjects gave their written informed consent prior to the study. This protocol was approved by the Research Ethics Committee of the University of Guerrero (registration number 012/2013).

### 2.2. Anthropometric and Clinical Measurements

Body weight was determined in light clothes and without shoes, using a Tanita body composition monitor (Tanita TBF-300 GS), and the height was measured to the nearest 0.1 cm using a stadiometer (Seca, Hamburg, Germany). From these measurements, BMI was calculated (BMI = weight/height^2^, kg/m^2^). Subjects were classified by BMI, obese ≥ 30 kg/m^2^ and normal-weight < 24.9 kg/m^2^, and by obesity class based on the criteria by the World Health Organization [32]. The body circumferences were measured with an anthropometric tape accurately within ±0.1 cm (Seca, 201, Hamburg, Germany). Blood pressure was measured in the sitting position with an automatic sphygmomanometer (OMRON) on the left arm after 10 min rest. The systolic blood pressure (SBP) and diastolic blood pressure (DBP) were calculated from two readings with a minimal interval of 10 min.

### 2.3. Laboratory Measurements

A venous blood sample of 5 mL was obtained from each subject after at least 12-hour fasting. Biochemical parameters, such as total cholesterol, HDL-cholesterol (HDL-C), LDL-cholesterol (LDL-C), triglycerides (TG), and fasting glucose levels, were analyzed immediately by enzymatic colorimetric methods with commercially available kits (Spinreact). The determination of MIF serum levels was performed by a commercial kit (LEGEND MAX Human Active MIF ELISA Kit, BioLegend) according to manufacturer's instructions. The MIF assay sensitivity was 17.4 ± 9.2 pg/mL. The criterion for the diagnosis of metabolic syndrome was based on the National Cholesterol Education Program Adult Treatment Panel III (NCEP ATP III) [33].

### 2.4. Genotyping of −794 CATT_5–8_ and −173 G>C MIF Polymorphisms

Genomic DNA was extracted from peripheral blood leukocytes and stored at −20°C until analysis. The −794 CATT_5–8_ *MIF* polymorphism was analyzed by conventional polymerase chain reaction (PCR) in a Thermal Cycler (Techne TC-412) using the following primers: 5′-TGT CCT CTT CCT GCT ATG TC-3′ (Forward) and 5′-CAC TAA TGG TAA ACT CGG GG-3′ (Reverse). Cycling conditions were as follows: initial denaturing at 94°C for 5 min followed by 30 cycles of 30 s at 94°C, 30 s at 60°C, and 30 s at 72°C and then a final extension of 5 min at 72°C. Amplification products were visualized after electrophoresis on an 8% polyacrylamide gel stained with 2% AgNO_3_. Fragments of 129, 133, 137, and 141 bp represented the −794 CATT_5_, −794 CATT_6_, −794 CATT_7_, and −794 CATT_8_ alleles, respectively.

The −173 G>C* MIF* polymorphism was genotyped by polymerase chain reaction and restriction fragment length polymorphism (PCR-RFLP). Amplification of a 366 bp fragment was completed using the previously reported primers [34]; 28 cycles and an annealing temperature of 60°C were used. The obtained fragment was digested with* Alu I* restriction endonuclease (New England Biolabs, Ipswich, MA, USA) by overnight incubation at 37°C. Finally, the digestion was resolved on a 6% polyacrylamide gel stained with 2% AgNO_3_. The −173G allele resulted in 268 and 98 bp fragments while the −173C allele was identified by 206, 98, and 62 bp fragments.

### 2.5. MIF Expression Analysis

Peripheral blood was collected in EDTA blood collection tubes (BD Vaccutainer, NJ, USA). Immediately after blood drawing (<1 h), total leucocytes were isolated using dextran reagent (USB Corporation, Cleveland, OH, USA), and the total RNA was obtained using Trizol reagent (Life Technologies) according to the Chomiczyki and Sacchi method [35]. The RNA concentration and purity were determined by spectrophotometry (NanoDrop 2000C, Thermo Scientific). The cDNA was synthesized from 1 *μ*g of total RNA, using oligodT and reverse transcription reagents as indicated by the manufacturer (Promega Corporation, USA). The cDNA samples were stored at −80°C until the real-time PCR assays. The MIF and glyceraldehyde 3-phosphate dehydrogenase (GAPDH) expression was quantified using TaqMan probes and all samples were run in triplicate using the conditions indicated in the TaqMan Gene Expression Assay protocol in a Light Cycler Nano System (Roche Applied Science). Relative gene expression levels were calculated using the 2^−ΔΔCt^ method (expressed as relative expression units), after validating similar reaction efficiencies of the interest gene (MIF) and the reference gene GAPDH by running serial dilutions of both genes [36].

### 2.6. Statistical Analysis

Data analysis was performed using STATA software (v.11.0) and GraphPad Prism 5 software. Differences in characteristics between groups were analyzed using the chi-square test for categorical variables (data presented as percentages), Student's *t*-test for parametric variables (data presented as mean ± SD), and Mann-Whitney *U*-test for nonparametric variables (data presented as median and 5th to 95th percentiles). The Hardy-Weinberg equilibrium test and genotype and allele frequencies were calculated by the chi-square test. *P* < 0.05 was considered statistically significant.

## 3. Results

### 3.1. Anthropometric and Biochemical Characteristics

As expected, obese subjects had higher body weight, BMI, waist circumference, hip circumference, and waist-hip ratio, as well as glucose, total cholesterol, triglycerides, and LDL-C levels than normal-weight subjects (*P* < 0.05). There were no significant differences in age, gender, or HDL-C levels between groups (*P* > 0.05) (data not shown).

Anthropometric and biochemical characteristics as well as metabolic abnormalities of study subjects according to gender are shown in Table 1. In both groups of normal-weight and obese subjects, body weight, height, waist circumference, waist-hip ratio, and systolic blood pressure parameters were higher in men than in women (*P* < 0.05), whereas men with normal weight had low HDL-C levels and body fat mass (*P* < 0.05) and obese men had high TG levels (*P* = 0.008) compared with the women from each respective group.

Table 1 also shows the prevalence of metabolic syndrome and its components, where we found in the normal-weight group higher prevalence of hypertension (12% versus 2%, *P* = 0.010) and hypercholesterolemia (20% versus 6%, *P* = 0.013) in men than in women. In the obese group, the prevalence of hypertension (41% versus 16%, *P* = 0.005), impaired fasting glucose (8% versus 0%, *P* = 0.04), hypertriglyceridemia (51% versus 25%, *P* = 0.009), and metabolic syndrome was higher in men than in women (49% versus 28%, *P* = 0.032).

### 3.2. Distribution of −794 CATT_5–8_ and −173 G>C MIF Polymorphisms

Both* MIF* promoter polymorphisms analyzed were in Hardy-Weinberg equilibrium in the control group (−794 CATT_5–8_,  *P* = 0.88 and −173 G>C, *P* = 0.44). The distributions of −794 CATT_5–8_ and −173 G>C* MIF* polymorphisms in normal-weight and obese subjects are shown in Table 2. The comparative analysis of genotype and allele frequencies of −794 CATT_5–8_ and −173 G>C* MIF* polymorphisms between groups did not show significant differences. We also compared the clinical and biochemical variables by genotypes of both* MIF* polymorphisms, but we did not observe significant differences (data not shown). Additionally, we performed haplotype analyses of both polymorphisms considering the following combinations: 5G, 6G, and 7C. The estimated frequencies of the 5G, 6G, and 7C haplotypes were 14%, 48%, and 38%, respectively, in the total population (data not shown).

### 3.3. Relationship of MIF Promoter Polymorphisms with Its Expression in the Studied Groups

**Figure 1:**
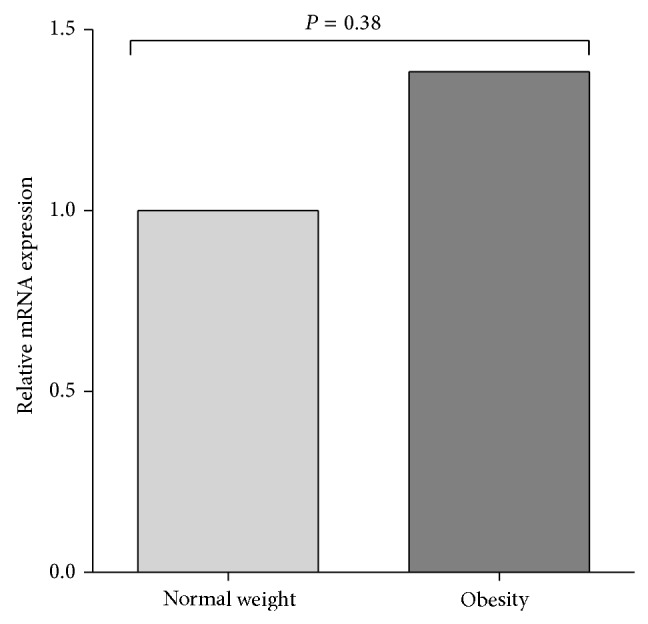
Relative MIF mRNA expression in normal-weight and obese subjects. Note that the obese subjects had a modest increase in MIF mRNA expression when compared with normal-weight subjects. Relative expression analysis was performed using the 2^−ΔΔCt^ method and* GAPDH* as the reference gene. Comparison among groups was performed using Mann-Whitney *U*-test; *P* < 0.05.

**Figure 2:**
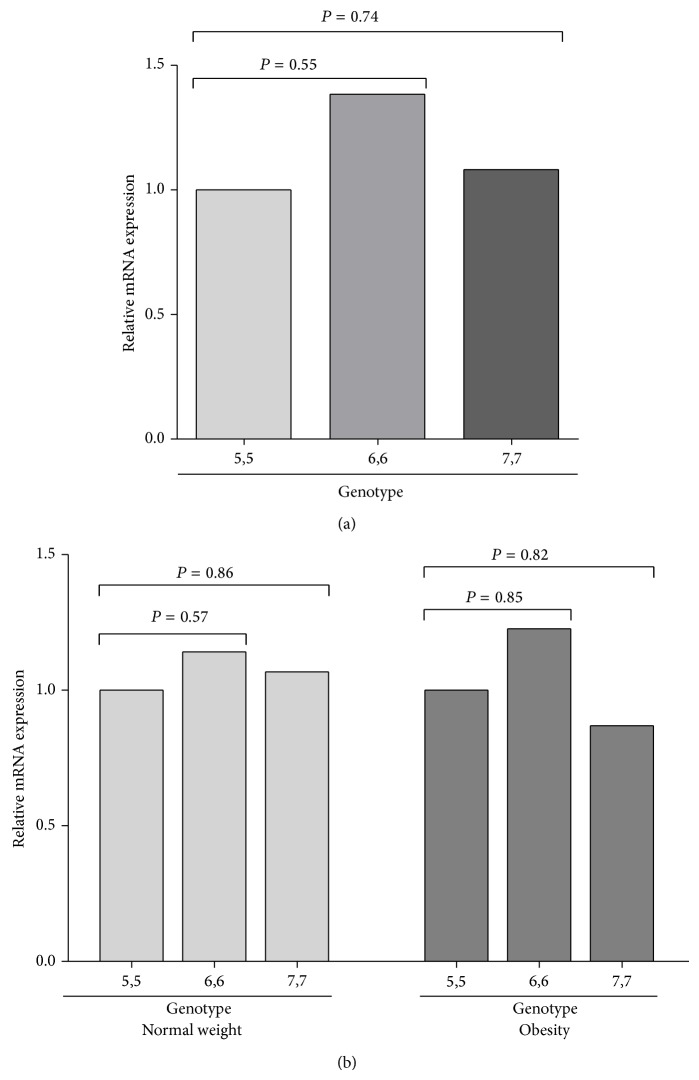
Relative MIF mRNA expression by −794 CATT_5–8_ *MIF* (rs5844572) genotypes in normal-weight and obese subjects. (a) The slightly high MIF mRNA expression was observed in the 6,6 carriers, while the 5,5 carriers had lower expression in the total population. (b) The modest increase in MIF mRNA expression was observed in the 6,6 carriers in both groups, while the 7,7 carriers had lower expression in the obese group. Relative expression analysis was performed using the 2^−ΔΔCt^ method, using* GAPDH* as the reference gene. Comparison among groups was performed using Mann-Whitney *U*-test; *P* < 0.05.

**Figure 3:**
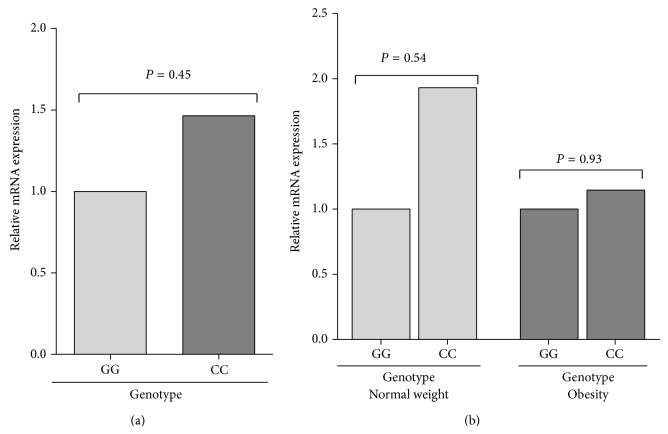
Relative MIF mRNA expression by −173 G>C* MIF* (rs755622) genotypes in normal-weight and obese subjects. (a) The slightly high MIF mRNA expression was observed in the CC carriers in the total population; (b) the GG carriers had lowest expression in both groups. Relative expression analysis was performed using the 2^−ΔΔCt^ method, using* GAPDH* as the reference gene. Comparison among groups was performed using Mann-Whitney *U*-test; *P* < 0.05.

**Figure 4:**
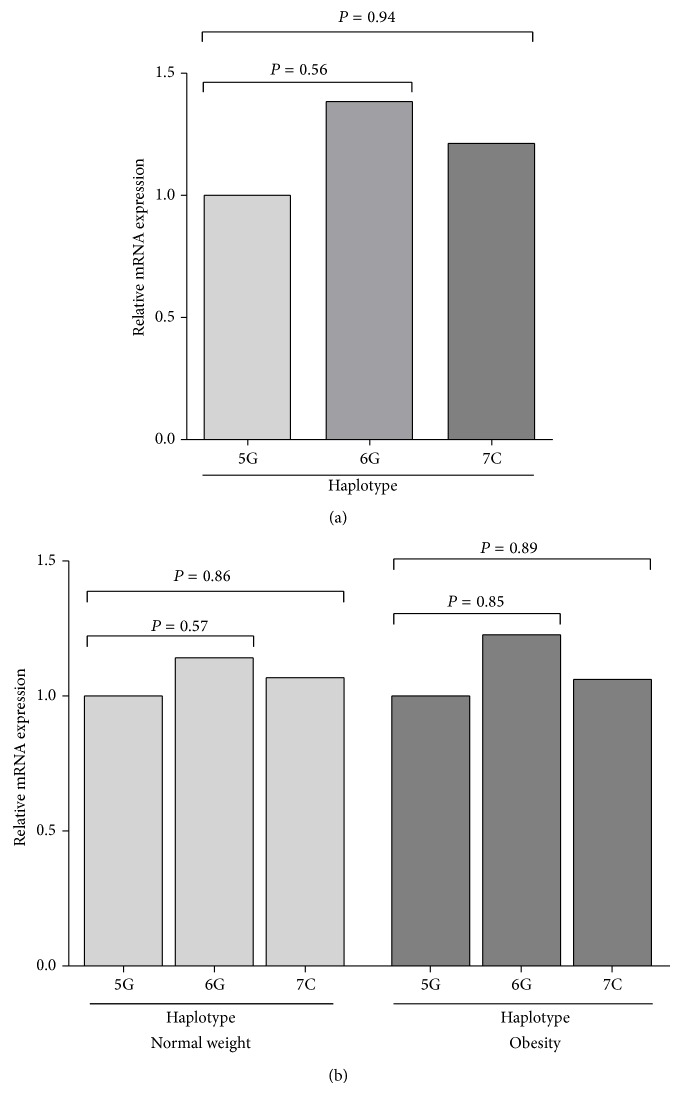
Relative MIF mRNA expression by −794 CATT_5–8_ (rs5844572) and −173 G>C* MIF* (rs755622) haplotype in normal-weight and obese subjects. (a) The slightly higher MIF mRNA expression was observed in the 6G carriers, while the 5G carriers had a low expression in the total population. (b) The modest increase in MIF mRNA expression was observed in the 6G carriers in both groups. Relative expression analysis was performed using the 2^−ΔΔCt^ method, using* GAPDH* as the reference gene. Comparison among groups was performed using Mann-Whitney *U*-test; *P* < 0.05.

Relative MIF mRNA expression in total leucocytes was slightly higher in obese subjects than in normal-weight subjects (1.38-fold) (Figure 1). To investigate the functional impact of both polymorphisms, the quantitative MIF mRNA expression among the different genotypes for both polymorphisms was analyzed. When we analyzed the expression according to the STR −794 CATT_5–8_ *MIF*, we found that carriers of the 6,6 genotype had slightly higher expression in comparison to the 7,7 genotype, and the latter with respect to the 5,5 genotype, in the total population (1.38 > 1.08 > 1) (Figure 2(a)). Similarly, when we compared the expression by groups, a modest increase of MIF mRNA expression was observed in the 6,6 carriers in both groups, while the 7,7 carriers had a low expression in the obese group. Additionally, the 6,6 obese carriers expressed slightly higher mRNA expression than normal-weight 6,6 carriers (Figure 2(b)). Carriers of −173 C/C genotype had a slightly higher expression than carriers of the G/G genotype in the total population (1.47 > 1) (Figure 3(a)). When we compared the expression by groups, a modest increase of MIF mRNA expression was observed in the carriers of the C/C genotype compared to the G/G genotype in both groups (Figure 3(b)). To analyze the combined effect of −794 CATT_5–8_ and −173 G>C* MIF* polymorphisms on the MIF mRNA expression, we analyzed the expression according to 5G, 6G, and 7C haplotypes. We found that the carriers of the 6G haplotype had the highest expression in comparison to the 7C haplotype, and the latter with respect to the 5G haplotype in the total population (1.38 > 1.21 > 1), although it was not significant (Figure 4(a)). Similarly, in both groups, the carriers of the 6G haplotype had high MIF mRNA expression (Figure 4(b)).

### 3.4. Serum MIF Levels and MIF Promoter Polymorphisms

**Figure 5:**
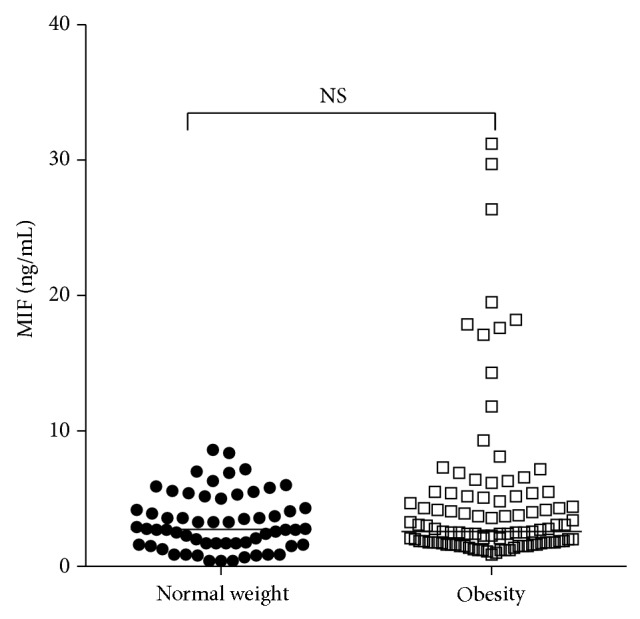
MIF serum levels by study groups. Data expressed as median and percentiles (p5–p95). Mann-Whitney *U*-test. NS: nonsignificant.

We analyzed MIF serum levels in obese and normal-weight subjects, but we did not find significant differences between both groups (*P* = 0.44) (Figure 5). When MIF serum levels were analyzed according to the −794 CATT_5–8_ and −173 G>C* MIF* polymorphisms, we did not observe significant differences (data not shown). Furthermore, a correlation analysis between MIF serum levels and measures of central adiposity was performed. In both groups of normal-weight and obese subjects, we did not observe a positive correlation between MIF serum levels with body weight, BMI, waist and hip circumferences, and waist-hip ratio (*P* > 0.05) (data not shown).

## 4. Discussion

This study shows that the MIF mRNA expression in total leucocytes is slightly increased in obese subjects when compared with the normal-weight group. However, we did not find a significant association between −794 CATT_5–8_ and −173 G>C* MIF* polymorphisms with MIF mRNA expression in young obese subjects.

The 6,6 genotype frequency for −794 CATT_5–8_ polymorphism and the frequency of the G allele for −173 G>C polymorphism were similar to previous studies in Mexican Mestizos from western Mexico populations [15, 27, 28, 37]. Conversely, for −794 CATT_5–8_ *MIF* polymorphism, the 5,6 genotype was the most frequent in a Japanese population [29] and, for the SNP −173 G>C, the C allele was the most frequent in Caucasian patients with psoriatic arthritis [38]. In the case of the −794 CATT_5–8_ *MIF* polymorphism, we did not observe the presence of genotypes with the −794 CATT_8_ high-expression allele which was reported as low frequency (1%) in Mexican Mestizo patients with RA and 0.4% in Japanese subjects [27, 29]. These differences may be attributed to the sample size and the inclusion criteria in each study, as well as to the racial influence among populations with different ethnic origin, thus conferring a greater genetic diversity in the distribution of these and other polymorphisms [39, 40].

Several studies have reported the relative MIF mRNA expression in obese subjects. Dandona and coworkers showed that MIF mRNA expression in mononuclear cells is significantly increased in obese patients compared to the control group and is related with plasma free fatty acids (FFA) concentrations and BMI but not MIF plasma concentrations or HOMA-index [18]. In another study, increased MIF mRNA expression in mononuclear cells was observed in obese subjects and correlated with BMI [41]. Our results suggest the involvement of MIF in the pathophysiology of obesity and its relationship with metabolic comorbidities.

Very few studies have reported the relationship between* MIF* gene polymorphisms and obesity. In 2006, Sakaue and coworkers found that −794 CATT_5–8_ *MIF* polymorphism was associated with obesity in a Japanese population [29]. In another study, the 6,7 genotype of the MIF −794 CATT_5–8_ polymorphism was associated with susceptibility to acute coronary syndrome in a western Mexican population [37]. To our knowledge, this is the first study that reports the relationship between* MIF* gene polymorphisms and MIF mRNA expression in obese young subjects. For the −794 CATT_5–8_ polymorphism, carriers of the 6,6 genotype had slightly high MIF mRNA expression in comparison to the other genotypes in obese and nonobese subjects. Besides, obese 6,6 carriers expressed high mRNA in comparison with the normal-weight 6,6 carriers, although it was not statistically significant. Previously, it has been reported that this repeat regulates basal and stimulus-induced transcriptional activity, which increases almost proportionally with repeat number in defined* in vitro* assay systems. Reporter gene assays have demonstrated that the CATT_5_ allele has the lowest basal level and stimulated MIF promoter activity compared to the CATT_6_ and CATT_7_ allele* in vitro* [22, 42], but it is unknown which type of transcription factor regulates MIF expression through the promoter region containing the CATT repeat, while carriers of the CATT_7_ allele show high circulating MIF levels [24].

For the −173 G>C polymorphism, we found that the carriers of the C/C genotype had slightly high* MIF* mRNA expression in comparison to the G/G genotype, in obese and nonobese subjects, although it was not statistically significant. In reporter gene analyses, it has been shown that the −173 G>C polymorphism plays a role in the gene transcriptional regulation in a cell-type dependent manner in which the C allele promotes transcription in a human T lymphoblast cell line (CEMC7A), while the G allele favors transcription in a lung epithelial cell line (A549). These changes in expression could be due to differences in the transcription factor interaction with the MIF −173 element. Based on the promoter sequence analysis, AP-4 transcription factor is a particular candidate [23]. Furthermore, the C allele is associated with increased circulating MIF levels [23, 24]. These findings provide a biological support to the results of the present study.

As mentioned above, the two polymorphisms have genetic effects on promoter activity through interactions* in vitro*; therefore the functional impact of the polymorphism should be considered with respect to the haplotype. We found that the carriers of the 6G and 7C haplotypes had a modest increase in MIF mRNA expression in comparison to the 5G haplotype in the total population, but in obese subjects and controls the carriers of the 6G haplotype had a tendency to increase MIF mRNA expression. Allele 6 was found more frequently in our population and in other studies it has been identified as a high-expression allele together with alleles 7 and 8; therefore the increase in MIF expression can be attributed to allele 6. This finding is consistent with a previous study in a reporter gene assay, where it was shown that the 6G haplotype had the highest MIF promoter activity in the A549 epithelial cell line, suggesting functional importance of the MIF promoter haplotype in determining levels of MIF gene transcription [43]. Furthermore, in Caucasian and African American populations with SLE, the 7C haplotype is associated with high circulating MIF levels [26]. In addition, the −794 CATT_7_ and −173C polymorphisms were in linkage disequilibrium in a Mexican Mestizo population (*D*′ = 0.87, *P* < 0.001), which indicates that both alleles are segregated in block from one generation to another and may confer a similar risk [27].

Also, we did not find significant differences between MIF serum levels in both groups. However, others studies have shown increased MIF serum levels in subjects with obesity and type 2 diabetes. Dandona et al. reported a correlation between serum MIF levels and the body mass index (BMI), finding that obese subjects with an average BMI of 37.5 ± 4.9 kg/m^2^ had a significant higher fasting MIF concentration (2.8 ± 2.0 ng/mL) than lean control subjects (BMI 22.6 ± 3.4 kg/m^2^; 1.2 ± 0.6 ng/mL) [18]. Similarly, increased MIF serum levels were found in overweight adolescents compared with those of normal weight, and MIF concentrations were associated with markers of inflammation and obesity [20]. Other studies confirmed elevated MIF plasma levels in obese individuals compared to lean subjects [41, 44]. Also, the effect of some medications and the reduction of body weight on circulating MIF levels have been determined. In obese subjects with metformin treatment, an antidiabetes drug decreased MIF plasma concentrations of 2.3 ± 1.4 to 1.6 ± 1.2 ng/mL after an intervention of 6 weeks, and, after withdrawal of the drug, MIF levels returned to their initial value indicating a metformin-dependent effect [18]. In addition, morbidly obese subjects who participated in diet and physical activity based weight management programs showed a significant decrease in circulating MIF concentrations after weight loss of 14.4 kg [19]. In another weight loss program, weight reduction of 4.4 kg was achieved with a 67% decrease in circulating levels of MIF [45]. In contrast to these studies, however, morbid obese subjects with BMI of 46.7 ± 5.8 kg/m^2^ show low plasma MIF levels (about 0.2 ± 0.4 ng/mL); after gastric restrictive surgery, the BMI decreased markedly (33 ± 4.8 kg/m^2^) while MIF concentrations remained low for 6 months during weight loss, after which they significantly increased to normal levels at 24 months postoperatively [46]. The relationship between obesity and MIF is not consistent and any causal relationship between obesity and MIF levels remains to be established [47]. Factors that may contribute to the variability in these studies include differences in gender, since MIF plasma levels are higher in males [30], the use of hormone replacement therapy (HRT), since women with HRT show 2-3-fold higher plasma MIF levels [19], circadian rhythm [48], and differences in* MIF* promoter genotypes leading to variations in promoter activity and MIF serum levels [22–24, 30]. However, the −794 CATT_5–8_ and −173 G>C* MIF* polymorphisms did not show significant differences with MIF serum levels in our study, results similar to those reported in Mexican Mestizo patients with RA [27], SLE [15], and psoriatic arthritis [28]; however, they were not able to replicate the association of* MIF* polymorphisms with MIF serum levels; this could be due to differences in the genetic structure of our population which may influence activity at the* MIF* gene locus.

Our results showed no correlation between mRNA expression and MIF serum levels, where the obese subjects had a slightly higher mRNA expression but not MIF serum levels in comparison with the normal-weight group. It is known that the mRNA expression of a particular gene is not always predictive of protein expression, and the correlation between the two can vary significantly [49]. There are several possible explanations for the differences between the mRNA and protein levels and these may not be mutually exclusive, including complex posttranscriptional mechanisms and variation in protein half-lives because cells can control the protein level in the cell through the rates of degradation or synthesis for a given protein, as well as by the different sensitivities in methodologies for detecting mRNA and protein expression [50]. These possibilities could explain our data. To understand the reasons for this discordance, the dynamic processes involved in synthesis and degradation of MIF must be investigated in future studies.

Finally, some limitations of our study should be considered such as the heterogeneity of comorbidities of our study subjects and, in reference to our small sample size, a greater obese group is desirable to improve the power of the study.

In summary, we did not find the evidence to support the relationship between obesity and* MIF* gene promoter polymorphisms with MIF mRNA expression in young obese subjects.

## Figures and Tables

**Table 1:** Clinical and biochemical characteristics by study group.

Variables	Normal-weight (*n* = 150)			Obese (*n* = 100)		
	Male (*n* = 56)	Female (*n* = 94)	*P* value	Male (*n* = 49)	Female (*n* = 51)	*P* value
Age (years)^c^	21 (18–25)	20 (18–26)	0.74	22 (19–28)	21 (18–25)	0.18
Weight (kg)^c^	62 (51.4–76.3)	52.4 (43.4–65.3)	<0.001	99.7 (82.7–126.7)	84.8 (74.3–108)	<0.001
Height (cm)^c^	169.5 (157–183)	156.5 (148.5–166)	<0.001	171 (160–182)	160 (151–171)	<0.001
BMI (kg/m^2^)^c^	21.7 (19.1–24.6)	22.2 (18.7–24.5)	0.86	33.9 (30–43.1)	33.8 (30–40.4)	0.90
Obesity^a^						0.88
Class I (30–34.9 kg/m^2^)	—	—	—	32 (65)	33 (65)	
Class II (35–39.9 kg/m^2^)	—	—	—	13 (27)	15 (29)	
Class III (≥40 kg/m^2^)	—	—	—	4 (8)	3 (6)	
Waist circumference (cm)^c^	79 (70–89)	75.2 (65–88)	0.0001	109 (99–131)	103 (89–116)	0.0002
Hip circumference (cm)^c^	94 (86–104)	93.4 (87–103)	0.48	115 (107–129)	118 (109–131)	0.07
Waist-hip ratio^b^	0.84 ± 0.05	0.80 ± 0.06	0.0003	0.94 ± 0.05	0.87 ± 0.07	<0.001
Body fat mass (%)	13.4 (8.2–20.5)	24 (15–32.4)	<0.001	33.9 (25.6–44.1)	42.1 (34.9–49.5)	<0.001
Body fat mass (kg)	8.5 (4.4–14.8)	12.4 (6.7–21)	<0.001	33.1 (23.9–55.2)	35.4 (25.9–52.7)	0.064
SBP (mmHg)^c^	115 (94–140)	103.5 (88–121)	<0.001	125 (103–141)	114 (98–134)	<0.001
DBP (mmHg)^b^	68.5 ± 8.3	68.3 ± 7.9	0.87	76.9 ± 10.7	73.8 ± 8.5	0.11
*Metabolic profile *						
Glucose (mg/dL)^c^	85.5 (70–104)	83 (68–98)	0.30	89 (76–114)	88 (71–103)	0.52
Cholesterol (mg/dL)^c^	156 (120–244)	152 (101–212)	0.11	167 (110–234)	171 (113–227)	0.43
Triglycerides (mg/dL)^c^	80.5 (40–188)	70 (42–167)	0.12	150 (63–420)	118 (51–287)	0.008
LDL-C (mg/dL)^c^	87.5 (33–207)	88 (37–158)	0.80	120 (72–184)	102 (50–187)	0.09
HDL-C (mg/dL)^c^	38 (26.5–58.8)	42 (25.3–65)	0.035	39 (28–60)	42 (31–63)	0.55
*Metabolic syndrome *						
Hypertension (≥130/85 mmHg)^a^			0.010			0.005
No	49 (88)	92 (98)		29 (59)	43 (84)	
Yes	7 (12)	2 (2)		20 (41)	8 (16)	
Glucose (>110 mg/dL)^a^			0.19			0.04
No	55 (98)	94 (100)		45 (92)	51 (100)	
Yes	1 (2)	0 (0)		4 (8)	0 (0)	
Hypercholesterolemia (≥200 mg/dL)^a^			0.013			0.67
No	45 (80)	88 (94)		41 (84)	41 (80)	
Yes	11 (20)	6 (6)		8 (16)	10 (20)	
Hypertriglyceridemia (≥150 mg/dL)^a^			0.050			0.009
No	45 (80)	85 (91)		24 (49)	38 (75)	
Yes	11 (20)	8 (9)		25 (51)	13 (25)	
Metabolic syndrome^a^			0.70			0.032
No	55 (98)	93 (99)		25 (51)	36 (72)	
Yes	1 (2)	1 (1)		24 (49)	14 (28)	

BMI: body mass index, SBP: systolic blood pressure, DBP: diastolic blood pressure, LDL-C: low density lipoprotein-cholesterol, and HDL-C: high density lipoprotein-cholesterol. ^a^Data presented as *n* and percentage. Chi-square test. ^b^Data presented as mean ± SD. Student's *t-*test. ^c^Data presented as median and 5th and 95th percentile. Mann-Whitney *U*-test.

**Table 2:** Genotype and allele frequencies of −794 CATT_5–8_ (rs5844572) and −173 G>C (rs755622) *MIF* polymorphisms in normal-weight and obese subjects.

Polymorphism	Normal-weight	Obese	*P* ^∗^ value
	*n* = 150 (%)	*n* = 100 (%)	
rs5844572			
Genotype			0.53
5,5	4 (3)	2 (2)	
5,6	20 (13)	18 (18)	
5,7	22 (15)	18 (18)	
6,6	48 (32)	22 (22)	
6,7	44 (29)	29 (29)	
7,7	12 (8)	11 (11)	
Allele			0.23
5	50 (17)	40 (20)	
6	160 (53)	91 (46)	
7	90 (30)	69 (35)	

rs755622			
Genotype			0.15
GG	68 (45)	37 (37)	
GC	69 (47)	47 (47)	
CC	13 (8)	16 (16)	
Allele			0.07
G	205 (68)	121 (61)	
C	95 (32)	79 (39)	

^∗^Chi-square test *χ* ^2^.
